# Hemoptysis due to Pulmonary Arteriovenous Malformation after Coil Embolization during Long-Term Follow-Up

**DOI:** 10.1155/2019/4506253

**Published:** 2019-10-20

**Authors:** Masashi Shimohira, Kenji Iwata, Kengo Ohta, Yusuke Sawada, Takeshi Hashimoto, Katsuhiro Okuda, Ryoichi Nakanishi, Yuta Shibamoto

**Affiliations:** ^1^Department of Radiology, Nagoya City University Graduate School of Medical Sciences, Nagoya 467-8601, Japan; ^2^Department of Radiology, Nagoya City Wet Medical Center, Nagoya 462-8508, Japan; ^3^Department of Radiology, Ichinomiya Municipal Hospital, Ichinomiya 491-0041, Japan; ^4^Department of Radiology, Koseikai Hospital, Toyohashi 440-0045, Japan; ^5^Department of Oncology, Immunology and Surgery, Nagoya City University Graduate School of Medical Sciences, Nagoya 467-8601, Japan

## Abstract

A 28-year-old man with a history of coil embolization of multiple pulmonary arteriovenous malformations presented with hemoptysis 11 years after initial embolization. A cavity lesion in the left upper lobe, which was accompanied by deformed coils and ground-glass opacity, was considered responsible for hemoptysis. Embolization of the bronchial artery was performed.

## 1. Introduction

Pulmonary arteriovenous malformations (PAVMs) are abnormal communications between the pulmonary arteries and veins without any intervening capillary beds, which cause hypoxemia, cyanosis, and dyspnea [1]. They are often associated with an autosomal dominant genetic disorder, hereditary hemorrhagic telangiectasia (HHT) [1–5]. This disorder is characterized by recurrent epistaxis, mucocutaneous telangiectasia, and visceral vascular involvement, including arteriovenous communications that may develop in any organ, especially the lung [1, 2]. PAVMs include no capillary filters, and as a result, small blood clots, bacteria, and occasional air or clotted blood within intravenous tubing can pass directly through a PAVM and into systemic circulation. Thus, neurologic complications, such as transient ischemic attack, stroke, and brain abscess, can occur. Therefore, treatment for PAVMs is justified even in asymptomatic cases. Previously, PAVM had been treated with pneumonectomy. From the late 1970s transcatheter embolization has been widely performed and is now considered the first-line therapy for this condition [6]. Thus, complications related to embolization are an important issue. We herein report an occurrence of an unusual complication related to embolization of PAVM.

## 2. Case Report

**Figure 1:**
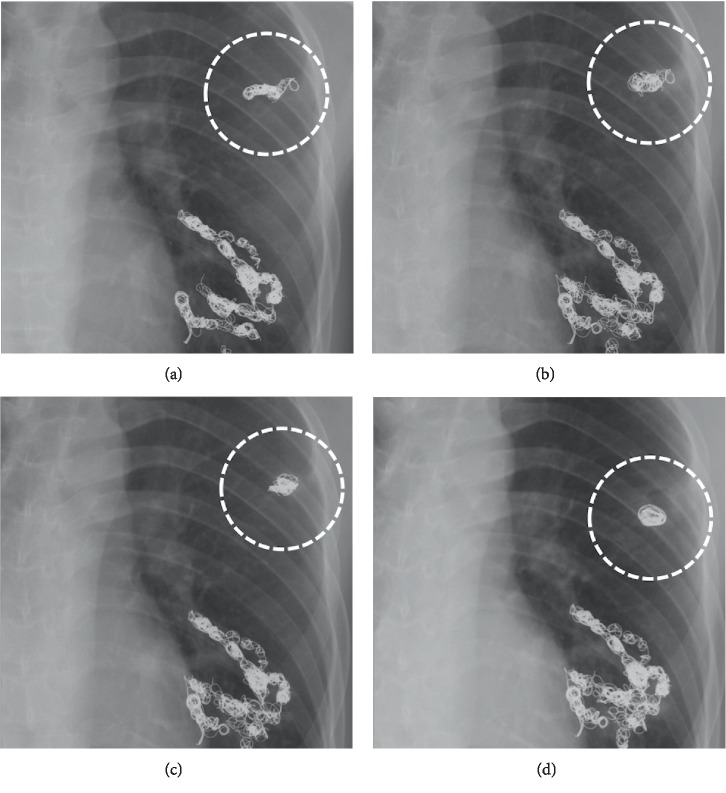
Chest radiograph images during follow-up showed that the coils used in embolization of the pulmonary arteriovenous malformation of the left upper lobe became deformed. (a) 11 years before, (b) 4 years before, (c) 2 years before, (d) latest examination.

**Figure 2:**
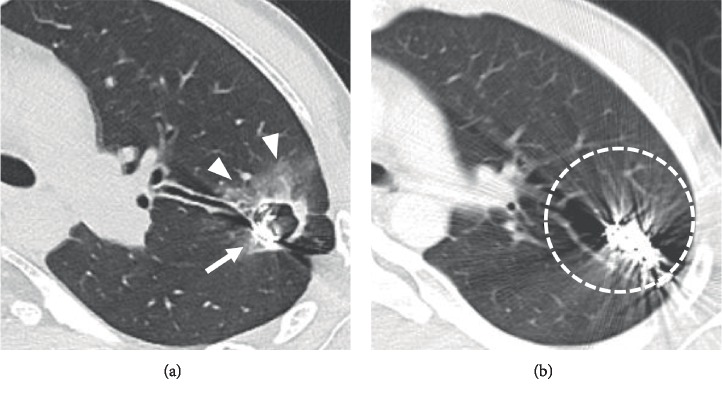
(a) CT shows a cavity lesion including the coils in the left upper lobe (arrow) and ground-glass opacity around the cavity lesion (arrow heads). We suspected that the ground-glass opacity might represent the source of the bleeding. (b) CT performed 10 years earlier shows no cavity lesions.

A 28-year-old man presented with hemoptysis. Eleven years earlier, he was diagnosed with HHT because he had multiple PAVMs, epistaxis, and positive family history. The genetic test for HHT was not performed, because it was not covered with medical insurance in our country. He underwent coil embolization of multiple PAVMs. During the follow-up, chest radiograph images showed that the coils in the left upper lobe became deformed (Figure 1). To evaluate the reason for hemoptysis, he underwent chest CT (Figure 2). We observed a cavity lesion at the left upper lobe, which also showed deformed coils and ground-glass opacity around the cavity lesion. In a previous CT, which was performed 10 years earlier, the cavity lesion and ground-glass opacity were not observed. We suspected that the ground-glass opacity represented the cause of the bleeding.

**Figure 3:**
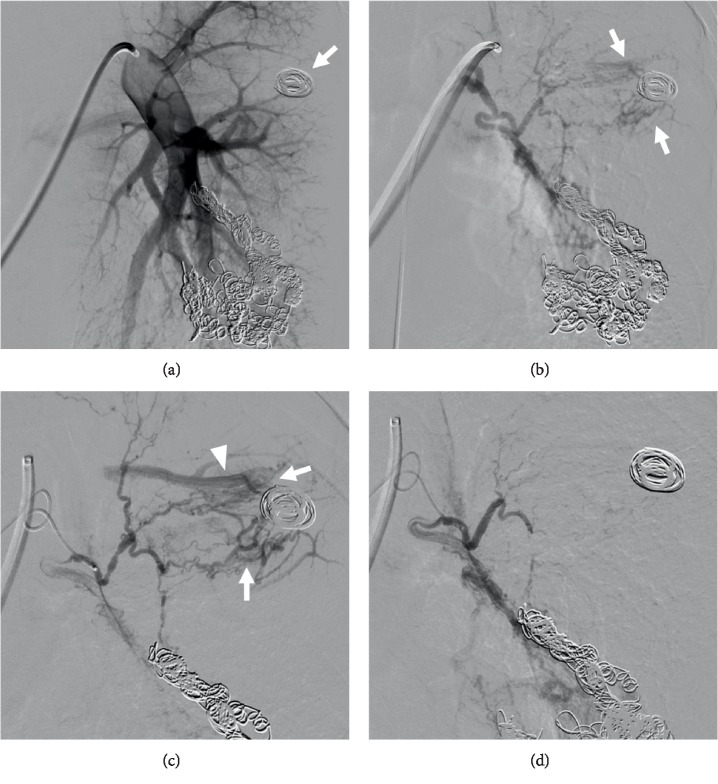
(a) Coils of the cavity lesion were confirmed (arrow), but pulmonary angiography showed no extravasation or hypervascular inflammatory parenchymal lesions. (b) Angiography of the left bronchial artery showed hypervascular inflammatory parenchymal lesions around the coils of the cavity lesion (arrows). Thus, the left bronchial artery was the vessel most likely to be responsible for the hemoptysis symptoms. (c) The microcatheter was advanced to the target branch of the left bronchial artery. Angiography showed hypervascular inflammatory parenchymal lesions around the coils of the cavity lesion (arrows) and bronchial–pulmonary artery shunt (arrow head). Embolization was performed using gelatin sponge. (d) Angiography of the left bronchial artery showed a complete occlusion of the target branch.

**Figure 4:**
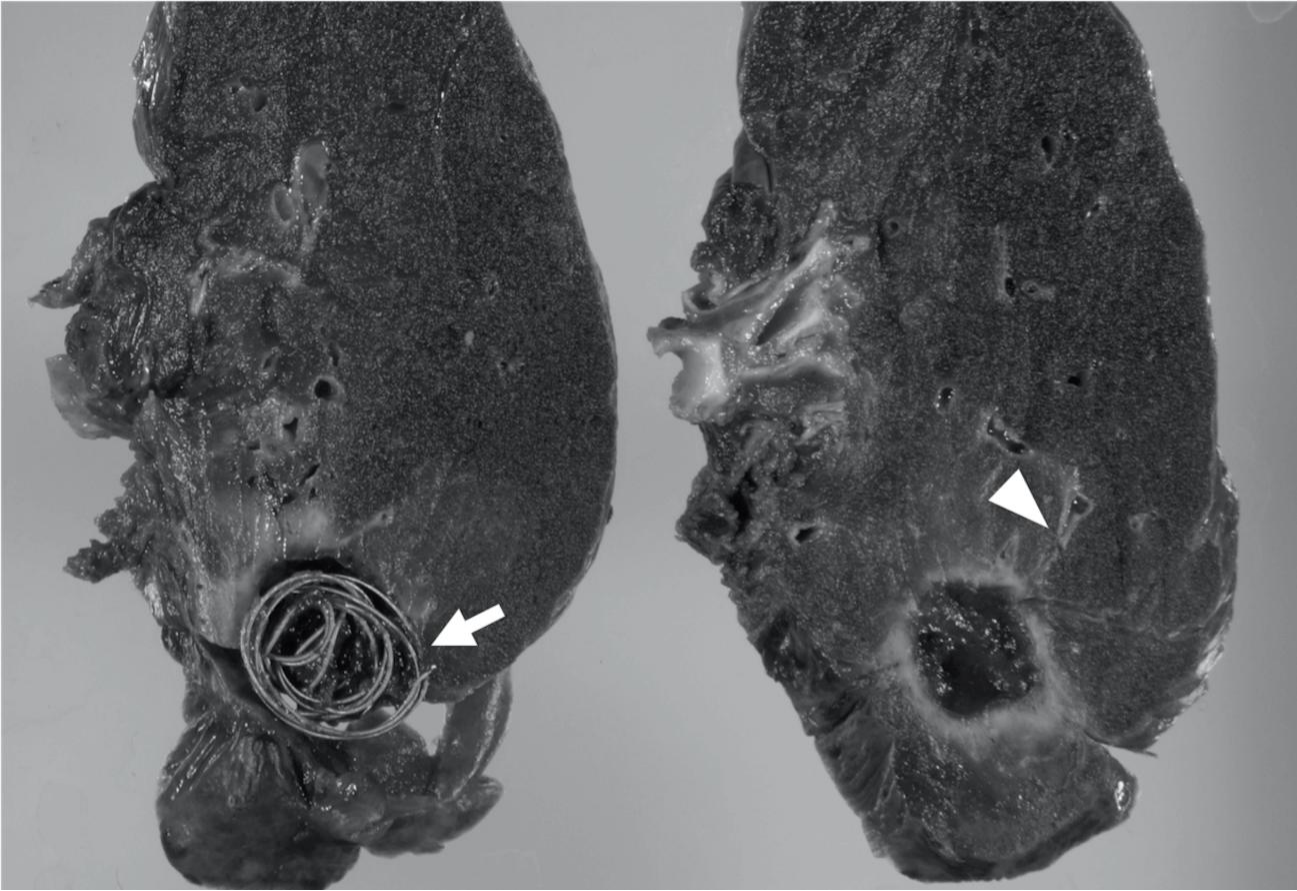
Photograph of the resected left upper lobe. The cavity lesion including placed coils was confirmed (arrow), and it was connected to the bronchus (arrow head).

Thereafter, angiography of both the left bronchial artery and pulmonary artery was performed to confirm which vessel was responsible for the symptoms (Figure 3). An 8-Fr. sheath was introduced into the right femoral vein, and a 4-Fr. sheath was placed at the right femoral artery. An 8-Fr. catheter (Optimo; Tokai Medical Products, Kasugai, Japan) was introduced into the pulmonary artery. Pulmonary angiography showed no extravasation or hypervascular inflammatory parenchymal lesions around the coils of the cavity lesion. Then, a 4-Fr. catheter (Broncho; Medikit, Tokyo, Japan) was placed into the left bronchial artery. Angiography showed hypervascular inflammatory parenchymal lesions around the coils of the cavity lesion. We concluded that the left bronchial artery was the vessel responsible for the hemoptysis. A microcatheter (Sniper 2 high-flow; Terumo, Tokyo, Japan) was advanced to the target branch of the left bronchial artery and embolization was performed using gelatin sponge. The reason for choosing gelatin sponge was as follows. We thought coils could only make proximal embolization, which might more readily allow recurrence of hemoptysis due to the development of other systemic arteries. In addition, polyvinyl alcohol and microspheres were not covered with medical insurance for hemoptysis in our country. Subsequent angiography of the left bronchial artery showed a complete occlusion of the target branch. After the procedure, hemoptysis markedly decreased, but a small amount of hemosputa remained. To complete the treatment, lobectomy of the left upper lobe was performed (Figure 4). Thereafter, hemoptysis disappeared during the two years of follow-up.

## 3. Discussion

Technical complications during embolization of PAVM have been reported to include PAVM perforation, migration of an embolic device into systemic circulation, air embolism, and coil reflux to the other pulmonary artery [4, 7]. PAVM perforation can cause hemoptysis or hemothorax. In addition, the migration of an embolic device into the systemic circulation and air embolism can induce cerebral ischemia. Therefore, a careful manipulation of catheters and embolic devices is necessary. Self-limiting pleurisy can occur within the first 48 hours of embolization and is sometimes accompanied by fever. These symptoms most likely result from localized pulmonary infarction caused by the occlusion of the normal branches of the pulmonary artery [4]. As a result, preserving normal pulmonary arteries is important during embolization. As a complication after embolization, it was reported that pulmonary hypertension occurred 10 days after the embolization of PAVMs in the presence of a left-to-right shunt resulting from hepatic arteriovenous malformations [8]. Thus, the presence of a left-to-right shunt should be checked before embolization of a PAVM. Furthermore, persistence is an important issue after successful embolization, and is attributable to recanalization, pulmonary-to-pulmonary reperfusion, incomplete primary treatment, and systemic-to-pulmonary reperfusion. Transient ischemic attacks have been reported as complications related to persistence [5]. Consequently, a follow-up examination is important to evaluate persistence.

Here, we report an unusual complication: hemoptysis from a cavity lesion of the lung including coils used in embolization, occurring 11 years after initial embolization. To our knowledge, there has been no previous report on cavity formation containing coils after embolization of PAVM. Images taken of the patient showed that the coils of the left upper lobe became deformed. We hypothesized that this change might have occurred as a result of an infection around the coils, and the coils subsequently migrating to the cavity. A foreign object prevented the healing of the infection and this resulted in chronic inflammation around the coils. The inflammation promoted angiogenesis in the cavity wall. These new vessels would likely have fragile walls and bleed easily, resulting in the hemoptysis that was observed in this case. After the embolization, hemoptysis markedly decreased, but a small amount of hemosputa remained. We considered that complete treatment was difficult to achieve by embolization alone because the coils were left in the cavity and could continue to be a source of inflammation. Thus, we decided to remove the coils completely by surgery. In the future, we propose that clinical symptoms and the structure of coils should be checked even in patients that have long-term follow-up.

## 4. Conclusions

Hemoptysis can occur due to chronic inflammation around the placed coils, and a careful follow-up is necessary.
